# A model for empowerment of nursing in Iran

**DOI:** 10.1186/1472-6963-5-24

**Published:** 2005-03-16

**Authors:** Mohsen Adib Hajbaghery, Mahvash Salsali

**Affiliations:** 1 Faculty of Nursing and Midwifery, Kashan University of Medical Sciences, Kashan, Iran; 2 Faculty of Nursing and Midwifery, Tehran University of Medical Sciences, Tehran, Iran

## Abstract

**Background:**

While the Iranian nursing profession tries to reach to its full capacity for participating in the maintenance of public health, its desire to develop is strongly influenced by cultural, economic, and religious factors. The concept of empowerment is frequently used in nursing and the health services, particularly in relation to the quality of care, since the mission of nursing is to provide safe and quality nursing care thereby enabling patients to achieve their maximum level of wellness. When considering the importance of nursing services in any health system, the 54th World Health Assembly recommended that programs be designed to strengthen and promote the nursing profession. Since empowerment is crucial to the role of nurses, a qualitative study was conducted and aimed at designing a model for empowering nurses in Iran.

**Methods:**

A grounded theory approach was used for analyzing the participants' experiences, their perceptions and the strategies affecting empowerment. Data collection was done through Semi-structured interviews and participant observation. Forty-four participants were interviewed and 12 sessions of observation were carried out.

**Results:**

Three main categories emerged from the data collected; these are "personal empowerment", "collective empowerment", and "the culture and structure of the organization." From the participants' perspective, empowerment is a dynamic process that results from mutual interaction between personal and collective traits of nurses as well as the culture and the structure of the organization. Impediments, such as power dynamics within the health care system hinder nurses from demonstrating that they possess the essential ingredients of empowerment.

**Conclusion:**

A model was designed for empowering the nursing profession in Iran. Implementing this model will not only define nursing roles, identify territories in the national healthcare system, but it will restructure nursing systems, sub-systems, and services. Currently no such model exists; therefore, restructuring of the nursing system, including its services, education and research subsystems is recommended.

## Background

Iran, a country of sixty eight million has a national health service which employs over 70,000 nursing personnel (including operating room technicians) who provide nursing care in general and specialty hospitals. Although the population of nurses is approximately one hundred and twenty thousand, many are unemployed. Among the unemployed are those who choose not to work after marriage. Consequently, Iran like other countries is faced with a nursing shortage. The impact of this nursing shortage lead nurses to work more than their required shift of 192 hours per month; with potentially 150 hours of overtime in some parts of the country. The role of nurses is unclear, for although most of them are employed in hospitals, yet few or none are in the role of Public Health Nursing. Additionally, because the people of Iran have a poor image of nursing, those who choose nursing as a profession do experience low self-esteem. The combination of poor image and questions regarding quality of patient care, one would find it hard to believe that these nurses are graduates with a Baccalaureate Science in Nursing (BSN) from either a nursing school or medical science university.

### Historical perspective of nursing in Iran

Historically, records of nursing in Iran before 1915 showed that nursing care was carried out by household women or servants. Hospitalized patients were also cared for by untrained personnel [1]. Because of this history, lack of basic education, low cultural status, and some religious limitations for women, nursing as a profession/career neither gained high standard nor recognition. However, 1915 is noted as the turning point for nursing in Iran. During that year the American Presbyterian Missionary Society (APMS) pioneered the training of a few nurses in a small missionary hospital. Subsequently, in 1916 a three-year nursing school was established in Tabriz. After 1916 there was a gradual increase of nursing schools across the country leading to a growing demand for nurse educators who were already scarce [2]. Because there was a lack of qualified nurse educators in Iran the World Health Organization (WHO) was appointed to assist in these schools. The Iranian faculty shortage was further supplemented with recruits from England and United States of America (USA). A two-year program of study was developed and the entry-level requirement for these schools was a minimum of nine years of general education [3]. This minimum requirement failed to attract a large number of prospective nursing students for nursing being predominantly female and in Iran women were prohibited to engage in any social activities that required close contact with men. Avoiding close contact with men is almost impossible in nursing, for healthcare system is not designed to be all male or female and therefore posed a threat for nursing schools and the recruitment of prospective nursing students.

Following the Second World War, Iran began gaining momentum for advancing nursing as a profession. The Princess Ashraf School of Nursing was established with the appointment of nursing faculty from England and offered a three-year program where the admission requirement was a high school diploma. Then, in 1943 The Iranian Nursing Association (INA) was formed by a group of Iranian nurses who were educated abroad and returned to the country [2]. Next, in 1952, a nursing division was established in the Ministry of Health and this was the first time nursing was officially recognized by the government and became part of its structure. The first university program for obtaining a BSN began in October 1967 at Shiraz University [2,3].

The increasing demand for healthcare in Iran forced the stakeholders of nursing education to develop new initiatives to meet the demand. As a result, in 1975 The Ministry of Sciences approved the Associate Degree of nursing program (ADN). Thereafter, nursing education in Iran was on the move, there were ADN, and BSN programs with a Masters of Science in nursing (MSN) program gradually developing. Although nursing education was growing, the numbers of graduates were still inadequate to meet Iran's demand for healthcare. For example, between 1915–1979 a total of 8,546 nurses graduated to provide healthcare services for a 30 million population. Because fully qualified nurses remained few in numbers, the alternative was to complement the nursing care in hospitals with auxiliary nursing personnel.

In 1979 when the Islamic revolution took place, major social and cultural changes occurred. These changes not only impacted the health care system, but nursing services too. Previously, the majority of nurses were female and they were caring for both men and women. However, as a result of the Islamic revolution, the government decided that nursing school admissions constitute 50 percent males, with the belief that men should care for men and that women must be separate from men while they engage in social activities.

Despite all the positive changes, bridging the gap of the nursing shortage continued to be a challenge, for new issues continued to emerge and there were no easy solutions or "quick fixes". Some of the issues that emerged were, increased birth rate causing an increased population, and the start of the imposed war between Iraq and Iran which only proliferated Iran's nursing shortage. Concurrently, the Iranian government decided to re-structure the Ministry of Health where it was re-named The Ministry of Health and Medical Education. Under this ministry new institutions of medical and nursing education were established and some existing institutions expanded. As a result, after the revolution 11,274 nurses were trained in the first decade, with an addition of 22,000 more nurses over the next 7 years [1-3]. Also, after the war, nurses' aides and ADN nurses were trained too. While providing educating the different types of nursing personnel narrowed the gap of the nursing shortage dilemma, it created role ambiguity and masked the criteria for "who is qualified to practice nursing".

To bring clarity to "who is qualified to practice nursing" the Ministry of Health and nurse leaders collaborated and wrote job descriptions for the different levels of nurses. However, these job descriptions were not fully implemented because of several obstacles such as, vague or to abstract job description, work overload, poor staffing in the hospitals, and lack of continuing education for nurses. Consequently, there were several overlaps among the varying roles for nursing personnel in hospitals.

By this time, admissions to nursing schools soared only to be faced with yet another hurdle to overcome. There were too few faculties and a dwindling number of institutions for clinical placement. As a result, many physicians and inexperienced nurses were assigned to faculty roles in nursing education. The mix of physicians and inexperience nurses drastically changed the philosophy and educational model for nursing education. Nursing education was primarily following the medical model. So nurses are taught the medical management of diseases and to follow physicians' orders without questioning; this educational model was not only oppressive, it silenced its participants and diminished their self-confidence. All this only exacerbated the difficulty of teaching professional nurses to be assertive and be in control of their practice by giving "voice" to their concerns.

Because the government did not recruit enough nurses during the war between Iraq and Iran, nurses had no choice but to work overtime and student nurses became part of the work force in order to compensate for the staffing shortage in hospitals. This shift in nursing trend converted nursing care to tasks and these nursing tasks soon became routine care in hospitals. It quickly became evident that new graduates were inefficient, so the MSN programs expanded with the main goal of preparing nurses for faculty roles. So, nurses were educated for roles according to nursing specialties such as, medical-surgical, psychiatric, community health, pediatric, and management, but even with this expansion roles were still not clearly delineated. Thus, many of the graduates from the MSN programs leaned towards nursing education rather than nursing practice. There seemed to be no end to Iran's nursing issues, it was time for a new approach.

So, after 30 years of fighting for state recognition, in 2002 Iranian Nursing Organization (INO) was approved by Iran's legislature and the INO established itself the same year. Now, the INO has the legal responsibility to represent all nurses in all sectors of nursing. Some of its key objectives are, improving the quality of patient care and developing standards for nursing practice. Even though this newly established organization could play a significant role in the development and empowerment of nurses, much time is needed to establish themselves as a governing body for nurses and they certainly have a tough job to shape the future of nurses and nursing in Iran. Since empowerment is crucial to the role of nurses, a qualitative study was conducted and aimed at designing a model for empowering nurses in Iran.

## Methods

A qualitative study was conducted using a grounded theory approach [4]. The purpose of study was to design a model for empowering nurses in Iran. The term grounded theory reflects the concept that theory emerging from this type of work is grounded in the data [5]. The researcher's purpose in using grounded theory is to explain a phenomenon within the social situation and to identify the inherent processes involved [6]. The grounded theory approach has been used in nursing research since 1970 and the studies have focused on nursing practice and education [7]. Empowerment is clearly a process rather than a static factor; therefore, a grounded-theory approach is the preferred methodology. In addition, this approach was selected because nurses' practice takes place in a multidisciplinary team and grounded theory focuses on identification, description, and explanation of interactional processes between and among individuals or groups within a given social context [8,9]. The research question is which processes influence empowering nurses and what is the appropriate model for empowerment of nurses in Iran. Data was collected by individual interviews which were audiotaped and transcribed and through observations which were recorded in field notes.

### Participants and data collection

The study comprised of 44 participants who are nurses in varying roles and settings, they are 12 nurses, 12 head nurses, two supervisors, three nurse managers (matrons), three nurse educators, three senior nursing directors (SND), two doctors and seven members of the INO. Purposive sampling was used at first and then continued with theoretical sampling according to the codes and categories as they emerged. Criteria for selection were nurses with more than five years of nursing experience who worked full-time in four large hospitals covered by the Ministry of Health and Medical Education in Tehran, Iran. Any nurse who met this requirement was considered a potential participant. Data collection began with staff nurses; after interviewing three nurses and coding the transcripts, the codes and categories that emerged were related to managerial support, organizational variables, and nursing education which led to the decision for the researcher to interview head nurses, supervisors and a few other key informants. Other key informants were higher-level managers, doctors, and nurse educators. Each interview session ranged from twenty minutes to three hours with an average of 115 minutes. Data was collected and analyzed over a six months period in 2003.

### Interviews

The main researcher contacted each of the potential participants to explain the objectives and the research questions. If the participant agreed to take part in the research, an interview was scheduled.

Participants were interviewed in a private room at the worksite using an individual semi-structured interview format and this was primarily the main method for data collection.

The interview guide consisted of open-ended questions to allow respondents to fully explain their own opinions, perceptions, and experiences. To start, each participant was asked to describe one of his/her own typical tour of duty; then to explain his/her own perceptions and experiences on "professional empowerment" and the "factors influencing it". For instance, he/she was asked, in your opinion, what is the meaning of empowerment in nursing?; Do you feel you are an empowered nurse?; can you describe some of your instances when you felt empowered?; which factors enhanced your experiences of empowerment? Tell me what are your thoughts on how can nurses and the nursing profession become empowered. Brief notes were made about the issues raised during the interview. Questions were asked later if these issues had not been spontaneously clarified. The interviews were transcribed verbatim and were analyzed consecutively.

### Observation

The main researcher conducted twelve sessions of participant observation in all four hospitals. Observations were conducted during the different shifts in emergency, medical, surgical and intensive care units and involved not only the nurses interviewed but also the other nurses present during the shift. To conduct the observation the researcher sat in a corner of the ward and either watched or followed individual nurses around. Even though the head nurse requested that the researcher do not participate in direct patient care, minimal assistance was given upon some nurses' request. The main focus of the participant observation was on nurses' interactions with their patients, colleagues, head nurses, supervisors and doctors. Emphasis was on nurses' participation on decisions related to patient care and practice settings. The brief notes taken at the time of observation were written up in detail on the same day. These detailed notes were used as data concurrently with the interviews.

### Data analysis

The data collection and analysis were done simultaneously according to the grounded theory approach. The interviews and observations data were analyzed concurrently using constant comparative method. Each interview was transcribed verbatim and analyzed before the next interview took place, so that each interview provided direction for the next. Following transcription, the tapes were replayed and notes were made onto the transcripts. Notes included comments such as tone of voice, recurrent themes, and the researcher's own thoughts and feelings about the nature and significance of the data.

Open, axial and selective coding were applied to data [6]. During open coding, each transcript was reviewed multiple times and codes were generated from the respondent's words and the researcher's constructs. For example, the code "managerial support" was generated by the researcher from a respondents' comments, "I think it is very important that we as nurses would be supported by our colleagues and managers. Powerful nursing owes support, it must be supported and the people in charge should support it." Codes that were found to be conceptually similar in nature or related in meaning were grouped in categories. The categories and codes from each interview were compared with other interviews in order to identify common links. Categories were related to their subcategories in axial coding. Coding was occurred around the axis of a category, linking categories at the level of properties and dimensions. Analytical tools include asking questions and making comparisons helped in finding the properties of each concept [6]. In this stage the structures of categories were related to the processes. For instance, the factors that contributed to nurses' feeling of collective empowerment or disempowerment were identified. The process of integrating and refining the theory occurred in selective coding. It is here that the main category "the culture and structure of the organization" was ascertained. Lastly, a model was developed to illustrate the important steps which are required for empowering nurses in Iran.

Although a variety of personnel at different levels were interviewed, the themes that arose were consistent across interviews. Interviewing stopped when data saturation occurred. Data saturation occurred when no more codes could be identified and the categories were "coherent". After data analysis each participant was given a full transcript of their coded interviews with a summary of the emergent themes to determine whether the codes and themes are true or matched their responses. Five faculty members also did peer checking on approximately 45% of the transcripts. The transcripts of interviews were given to each of the above persons and they followed the same process as above to arrive at core themes. There was a 90% or greater agreement between different raters. Results were also checked with some of the nurses who did not participate in the research and they confirmed the fitness of the results as well.

Sampling strategies allowed for maximum variation to occur and a vast range of views and perspectives to be considered. The researcher attempted to have precise documentation of the direction of the research and decisions made to save the "auditability" that will make it easier for other researchers to follow. Prolonged engagement with participants in the research environment allowed the researcher to gain participants' trust and better understanding of the research environment.

### Ethical considerations

Permission to conduct this study was through the ethics committee of Tehran University. Other ethical issues in this study involved the assurance of confidentiality and anonymity of the participants and their responses. All participants were informed of the purpose, design of the study, and that their participation was strictly voluntary. Participants signed a written consent to be in the study and permission was obtained from hospital directors and head nurses for the nurses to be interviewed and observed in the work setting.

## Results

Three main categories emerged from the data and each has 1–3 subcategories. These categories and their subcategories are representative of the main factors influencing the empowerment of nurses/nursing in Iran.

### Category 1: personal empowerment

According to the participants, personal empowerment is dependent on three variables, these are "having authority" and "professional self-confidence" for the "application of professional knowledge and skills." According to two nurses, "A powerful nurse is one who has good knowledge and can use it well" and "The power of a nurse depends on his knowledge and skills as well as his self-confidence in application of his knowledge in the provision of care for their clients." Furthermore, participants pointed out that the culture and structure of organization negatively impacts nurses' self-confidence and authority and it is one that emphasizes "physician centeredness". Two participants noted that "I must have the right to do nursing care based on my diagnosis, but I haven't this authority now," "a person can be powerful only when he/she can decide on his/her own." Others pointed out that "the public hasn't an appropriate view on the nursing profession," "nurses cannot provide their own services to the public directly and people go to the doctor first," "nurses are only expected to do the doctors orders," "their workloads are high," and "there isn't any system for nurses' continuing education." Collectively all these variables have a negative effect on nurses' self-confidence and minimized their ability to exercise authority or power in the practice setting.

### Category 2: collective empowerment

According to the participants "collective power" of nurses or the power of the profession of nursing comes from the interaction between two variables "supportive management" and "unity."

They considered the unity of nurses and coming together in nursing organizations as an efficient way to increase their professional power. Two participants commented: "to me the most important factor in the power and influence of a profession is the unity of its members". "We could be powerful when we are together and the best way for this is connecting to the nursing association, by this we can develop standards and regulations for our profession and these are the important prerequisites for supporting the nurses as professionals. These are prerequisites for determining our domain of professional authority."

The participants were aware that collective power brings them the possibility for obtaining better working condition as well as professional self-regulation. Also, collectively their voices are stronger and more influential. The latter would make it possible for them to develop standards for their profession and clearly define the scope of authority and responsibility of each nurse in his/her specific setting. Thereafter, these nurses will be able to apply their professional power in their practice settings to provide high-quality patient care. Nurses frequently stressed that supportive management will empower them. The participants' views and experiences on supportive management were categorized under the three subheadings of "provision of job related supports," "provision of financial support," and "provision of emotional support." However, a feeling of being unsupported was dominant among nurses. Shortage in the nursing workforce and lack of supplies such as sheets, dressing equipment and so on, prevented nurses from applying their professional knowledge and skills. Consequently, they were unable to meet their clients' needs, which give them a feeling of inadequacy and dis-empowerment.

### Category 3: the culture and structure of organization

The culture and structure of the health care system was another important factor that either facilitated or inhibited nurses' abilities to feel empowered. As participants indicated, these factors hindered the nurses' power in the patient care settings. This is true; when nurses are part of an organization that is "physician centered" they are almost extinct. One nurse manager noted "the nurses capabilities are not use appropriately because of some cultural and organizational factors". Data related to the cultural and organizational variables were categorized under two sub-headings "the public culture" and "organizational culture and structure."

### The public culture

Most of the nurses participating in this study mentioned the poor imagine of nursing being held by the Iranian people. Participants felt that the general public did not recognize the professionalism of nurses. One nurse commented in this regard: "the word nurse generalized to anyone who care for childes, sick or elderly, but not to a person who is expert in professional nursing care." Another nurses stated: "Because of the low social status and a poor public image involved with nursing, I always try to avoid conditions where people ask about my work, so I hate to say anything about it," "We are seen as doctors' handmaidens, for people goes to the doctors first, but they referred them to the hospitals, where nurses are."

The public status of physicians has affected the culture of health care organization. Inter-professional relationships have been affected by this culture and uneven power relations have developed in which the doctors have the last word. One of the participating doctors said: "the culture of health system doesn't regard nurses as professionals. There is an old and unscientific climate that induced a unilateral relationship between doctors and nurses. I think that many physicians don't know the real meaning of nursing."

### Organizational culture and structure

The belief that public and organizational culture have led to the development of a physician favored structure in the health care system was well articulated by the majority of participants. This is evident in the following quotes: "the health system is at hands of the physicians," "all of the top managers of health system and also in hospitals are the physicians," "nurses are only considered as tools for carrying out the doctors orders." The design of the nursing system was affected by this physician-centered structure. The participants mentioned three parts of this system which included nursing services, nursing education, and nursing research.

### The structure of nursing services

Classifying nursing services as in-patient settings was considered a barrier and limitation to the true potential and capability of the nursing profession. One participant's comment was "there are many nurses with a major in community health but they are not promised for working in the community." According to the participants, the root of this problem lies in the ambiguity of scope and standard of practice for nurses in the national healthcare system. One of the senior nurse directors who hold a position in the ministry of health shared: "we lacked a logic in our health system and also in our nursing system. So, the territory of nursing is not clear," Another participant emphasized that: "we are lacking a defined philosophy for nursing in Iran. This created structural obstacles that limited their power to implement their professional knowledge." It is obvious that there is an emergent need to clearly define the scope and standard of nursing practice in Iran, for only then can Iranian nurses practice as empowered professionals across the different areas within nursing. One of the senior nurses stated: "I believe that defining the mission and territory of nursing is the first step in the restructuring the nursing system." He believed that "we only offer some primary care within the hospitals, while we should integrate our services into the national health system. This is the best way for us to introduce our capabilities to the public." Also, this need is articulated by other nurses as evidenced in their statements "We have not clear professional rules and regulations;" "We don't really have a clear job description... one person's interpretation of what I should be doing could be different to mine... I would have some difficulties with my management over decisions that I would have made and they would say 'you should be doing that', and I would say 'I just can't'."

When the researcher asked the participants "what should be the first step in the process of empowerment of nursing in our country?" they responded, "We should define the personal and professional domain of nurses and their roles and position in health system." They believed that these should be done by the nursing organization. One of the senior nursing directors voiced "a group of specialists in the field of nursing from nursing unions, nursing schools, and the nursing office in the ministry of health should define the domain and roles of nursing...this is after such an important step that we could revise our nursing system and make it empowered so that nurses could implement their professional knowledge and skills for their clients at the level of hospitals and the community." The structure of in-patient nursing services was physician centered and routine-oriented. One of the supervisors stated: "...nothing could be done based on the nursing process...it is expected that nurses only obey orders, give the drugs, monitor the blood pressures ...but that they do not intervene independently." Inadequate staffing and having to perform non-nursing duties were felt to be disempowering and inimical to the recognition of nursing as a profession. These were highlighted as barriers to provide quality nursing care. "We've taken on every role. If something needs to be done and nobody else is going to do it I am compelled to it. There will be trouble if I don't do it" one nurse said.

### The structure of nursing education

There was great concern regarding the education system even though this plays an integral role in the process of empowerment. This concern originates from entry into nursing school as supported by the statement of one participant: "entrance examinations only measure the academic capabilities of volunteers but do not measure their compatibility to the nursing profession." Another concern is the curriculum content, it is highly theoretical and one nurse said: "...nurse educators think the best nurses are the nurses who have more medical information. They give them an extensive range of disease-related, pharmacological and physiological information, but don't spend even ten minutes on the nursing care in a class of two hours."

Role models also played a significant role in the weakness of nurses. It seems nurse educators doubt their own confidence, competency, and autonomy and were ineffective role models for students. An experienced nurse educator believed that "due to inexperience and freshness of most of the nurse educators, they lacked self confidence and could not educate a good new nursing generation." A philosophy of nursing education was considered absent and the question at large was "what are the guidelines for nursing education"? Therefore, "there is no relationship between nursing and clinical setting and the clinical setting is inappropriate for students' clinical placement" as one senior nurse director said. As a result of all these factors, nursing schools will continue to graduate dis-empowered nurses.

Providing continuing education was judged critical for nurses to maintain competency in the clinical setting and to become life-long learners so they can develop confidence in "giving voice" to continually improve nursing practice and build a community of empowered professionals. However, low staffing and lack of staff development resources by 'the Ministry of Health and Medical Education' only blocked the cycle of empowerment. As one participant pointed out: "I think continuing education is empowering. People who are educated are more empowered to carry out their work, but the ministry of health doesn't support in-service education for nurses".

### The structure of nursing research

Research utilization or the implementation of evidence-based practice was difficult for nurses for a variety of reasons. These are, the traditional structure of hospitals, poor quality of education, lack of continuing education, heavy workloads, no time, no mentoring and/or training in designing and conducting research, lack of financial resources, poorly defined nursing roles, lack of team work, and no opportunities for interdisciplinary relationships. These barriers are evident in the following quote of a nurse educator: "the research findings don't use in our nursing practice at all. We are two groups in nursing. One group is teachers and mainly teaches in nursing schools, another group is clinical nurses who are very busy and are also not educated for doing research... some of nurse educators also conduct researches not to be used in practice but only with the purpose of their promotion."

## Presenting the model of empowerment

**Figure 1:**
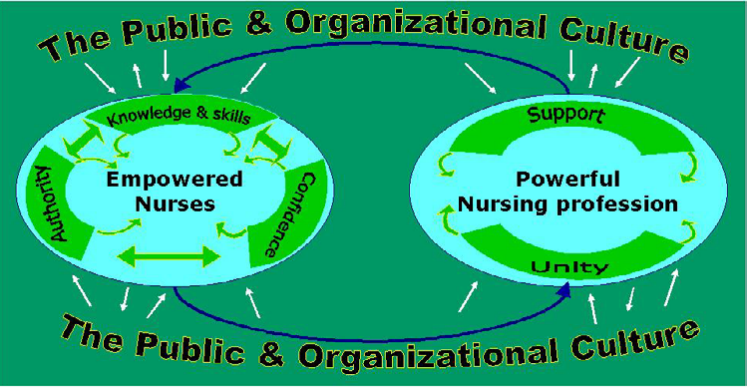
Relationship between personal and professional empowerment

Professional empowerment is a dynamic process, which occurs through interaction at the personal, professional, cultural and organizational levels. On one hand, the existence of competent nurses (those who have a wide range of professional knowledge and skills, authority and self-confidence) is essential for empowerment, as presented in figure 1- and on the other hand, the power of the profession and the public's image of nursing can affect nurses' self-confidence for demonstrating their full capabilities.

Through our own knowledge, action, and behavior personal and professional power is created [10]. Those who build excellent interpersonal relationships by demonstrating their knowledge gain credibility and experience a sense of empowerment [11]. As previously indicated, empowerment of nurses has been negatively affected by a multitude of cultural and structural factors which negatively affected the nurses' professional self-confidence and superimposed the belief that nurses are subordinates and handmaids to physicians. This caused nurses role to be restricted only to hospital rather than expanding it to the community setting. Other structural factors such as heavy workloads, inadequate staffing, lack of evidence-based nursing, and the task-oriented nature of nursing, have impeded empowerment of nursing as a profession and the role of nurses specifically in the application of their professional knowledge and skills [12].

As our data indicated, there was great concern regarding the education system even though this plays an integral role in the process of empowerment/dis-empowerment. The negative effects of incompetent nurse educators, lack of appropriate educational resources, oppressive methods of education, and using nursing students as part of the work force have also been confirmed by Adib hagbaghery et al.(2004); Espeland and Shanta (2001) and Chun-Heung (1997) [12-14].

**Figure 2:**
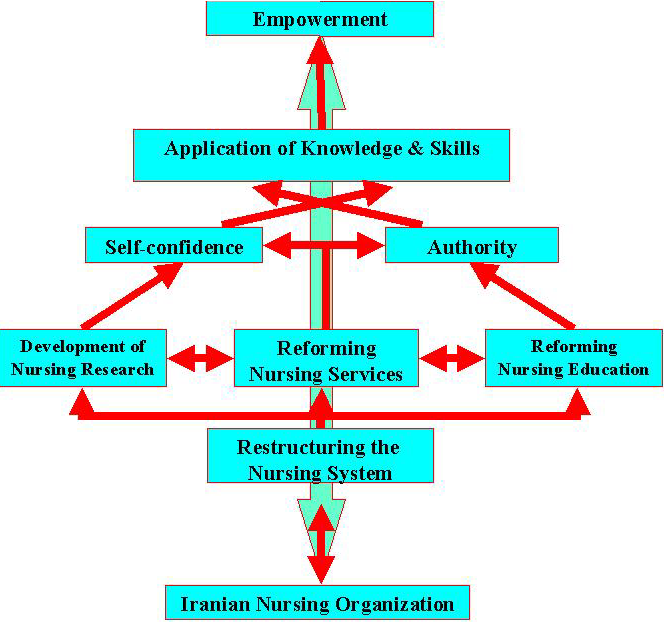
The model for empowerment of the nursing profession in Iran

After careful review, the data confirmed that "the culture and organizational structure" are the main variables affecting empowerment of nurses. Thus, a model of empowerment was developed and its emphasis is on re-designing the nursing systems. In this model, nursing system has three interrelated sub-systems; these are education, service, and research. Figure 2 represents the model and its major variables as well as the relationship between those variables influencing empowerment of nursing as profession.

### The goals of model

1. Strengthen the INO as a professional organization to represent nurses in the country

2. Re-design nursing system in hospital and community

3. Extend the implementation of comprehensive care in the country

4. Establish a national policy for nursing education

5. Revise and update the curriculum for the BSN nursing programs

6. Establish post-graduate education according to specialty and develop continuing education for nurses

7. Develop scope and standards to regulate nursing practice

8. Develop a valid and reliable system to administer licensure examination for nurses

It is imperative that these goals are achieved since it supports professional growth and empowerment of nurses in Iran.

As the model depicted, empowerment is a dynamic process, which results from interrelationship of personal and organizational factors. In other words, the process of empowerment requires changes not only in the structure of the organization but in the nurses' perception of themselves and their role. These changes will lead to transformation and the development of new perspective by the nurses not only of themselves but their organization too. This transformation will provide new lens through which individuals will seek new knowledge, and develop new problem solving skills, acquire a variety solutions that can be observed in their thinking, and actions. Nurses, then, will replace their subservient behavior and routine based nursing care with evidence based practice to support their nursing care actions, so having nurses to function at this level is necessary for the process of empowerment.

Reforming and the close inter-relationship of the three sub-systems are the cornerstone for restructuring the nursing system. Therefore, nursing education will produce competent and confident nurses; nursing services will support quality patient care and other subsystems; and nursing research will interact with other sub-systems to form an interdisciplinary team which will provide the knowledge and skills for conducting research that will not only identify issues but will provide creative solutions.

**Figure 3:**
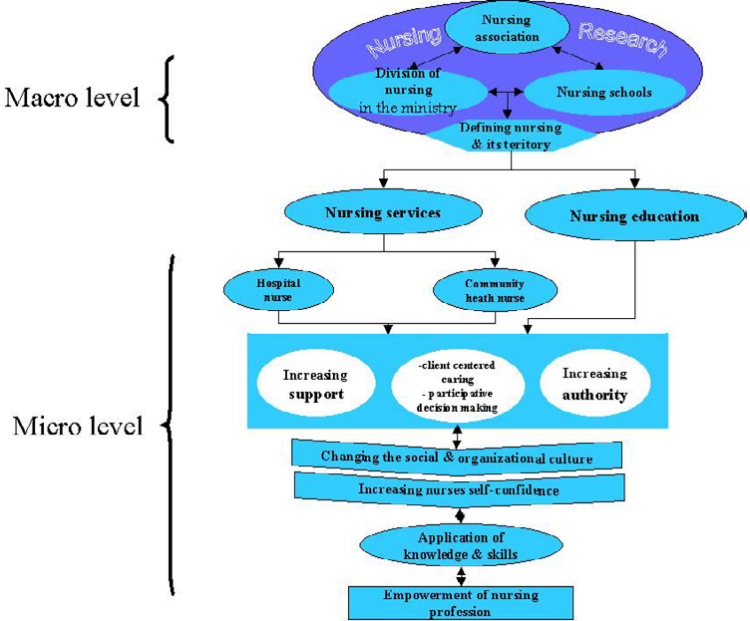
Steps in the model for empowerment of the nursing profession in Iran

Empowerment of nurses/nursing has been considered at both the micro and macro levels (fig. 3). At the macro level is the domain of nursing in the health system. As findings in this research have identified that nursing, its mission and its position have not been clearly defined in Iran. So, the first and most important step will be to define nursing, its mission and establish its position in the national healthcare system. Thus, the establishment of a committee named "the Scientific Committee for Defining Nursing and its Mission" (SCDNM) is recommended. The members of this committee will include members of the INO, nursing schools and the division of nursing in the Ministry of Health. Presently, these three parts are separate, but they will connect through their research subsystems for the execution of important mission such as:

- Defining nursing as a profession in the whole body of the national health system

- Declaring a philosophy of nursing and stating its mission and position within the national health system

- Designing clear job descriptions for nurses in general as well as in each specific setting

- Establishing standards, rules and regulations for the profession

- Establishing salary scale, standard of care, and nurse patient ratio, identifying staff competencies

- Developing and integrating the community nursing system in the body of the national health system

- Designing strategies for the development of nursing research and evidence based nursing

A review of the findings showed that, content and methods of nursing education along with the admission procedure need to be revised. Thus, the establishment of a committee named "the Committee for Revision in Nursing Education" (CRNE) was recommended. The committee consists of INO members, nursing schools, and the division of nursing in the Ministry of Health. The CRNE will develop an appropriate procedure for admission into nursing schools; evaluate and revise the content and process of nursing education- based on the approved criteria and the mission for the nursing profession in Iran.

The primary purpose of the revision was to improve the quality of nursing education so that when nurses graduate they are both competent and confident in their roles whether they are nurse generalist or specialists; they possess excellent inter-personal, critical thinking, and problem-solving skills which will enable them to identify their clients' healthcare needs both in hospital and the community. Establishing a system for continuing education and in-services for nurses was highly recommended.

The philosophy, mission, and position of nursing, will be the base for restructuring the nursing service. An important strategy will be to develop and integrate community health nursing in the national healthcare system thereby providing comprehensive health care. This initiative will extend nursing care at the community level and increasing the visibility of nursing. The increased visibility will allow the public to observe the role of nurses and their capabilities so that the public can acquire new perspectives and opinions regarding nurses/nursing and this will help regain nurses' self-confidence.

Another committee named "the Human Resources and Restructuring Committee" (HRRC) is also recommended for implementing the above strategy and restructuring the nursing system at the hospital level. The following tasks will be implemented:

- Evaluation of nurse-patient ratio and recruitment procedures

- Establishing standards for staffing levels and dividing them into three levels of Novice, Competent and Expert, according to their education, experience, and expertise

- Designing specific and clearly defined job descriptions for each level

- Allocating nurses in positions and on each nursing unit according to their level of expertise

- Fostering the staff nurses' professional growth by introducing a council system and developing a participative management style for nursing

These strategies will support nurses' professional growth but will provide opportunities for nurses to be involved in making decision regarding their professional practice.

Participating in the research and implementing its findings will empower nurses; they will have ownership in the changes they make. Empowered nurses will certainly make the health care system more effective and efficient. According to the results of this study, the traditional, typical structure, and processes of hospitals and healthcare systems prevented conducting and utilizing research in nursing. Hence, conducting nursing research and implementing research utilization will be re-enforced in nursing through the coordination and interaction between researchers and nursing services. They will strengthen the research structures and develop strategies for conducting future studies. The following actions were recommended:

- Adding nursing research to the curriculum of nursing

- Establishing a nursing research center in all nursing schools, hospitals, clinical-educational centers, and strengthen the existing centers

- Providing nurses with facilities to learn and conduct research projects

- Supporting nursing research by providing financial, informational, technical and management support including the provision of adequate manpower for nursing services

- Providing facilities for nursing faculties to conduct scientific seminars and conferences in nursing research.

These strategies can promote understanding of the research process and facilitate future nursing research studies. This will help nurses utilize the research findings and enhance the quality of nursing care and practice settings.

## Discussion

Nurses in this research frequently complained about lack of authority, some of them implied that they have the authority but do not use it due to some organizational and individual barriers such as heavy workloads, staff shortage, ambiguous job descriptions and the lack of self-confidence. These findings are confirmed with studies carried out by Laschinger et al. (2000), Fulton, (1997), Scott, Matthews and Corbely (2003) [15-17]. Reviewing the existing organizational structure, taking measures to balance medical, nursing and administration input to strategic planning and decision making, clarifying nurses' roles in different fields in the hospital and the public setting will all help to set professional boundaries and improve the interdisciplinary relations. These measures will help nurses exercise power and authority and empower them to make use of their knowledge and skills in practice. According to our findings, the culture and the structure of the organization along with lack of support and authority have decreased nurses' self-confidence. This finding has also confirmed by Madjar (1997) and Fulton (1997) [18,16]. Thus, restructuring the health system, particularly the nursing system based on its philosophy and mission will strengthen nurses' professional identity and self-confidence. Feeling of self-confidence is an essential factor for self-efficacy [19] and the process of empowerment. Restructuring of organization is an important way for increasing staffs' professional self-confidence [20,17]. Therefore, restructuring the nursing systems at all levels will empower and enhance nurses' professional self-confidence and allow them to deliver quality patient care.

As the results of this study indicated, application of professional knowledge and skills and improving the quality of health care are the most prominent outcomes from restructuring the nursing system. The publics' recognition and its view of nurses and the nursing profession will increase. This will enhance the nurses' professional identity, their self-confidence, and will certainly empower them for developing and demonstrating their professional capabilities. Extending nursing services at the community level, developing a participative management style, mobilizing active staff involvement in a councilor structure, and involving nurses in the process of decision-making will empower nurses and replace their routine-oriented style of delivering nursing care with decisions based on their professional knowledge and skills [21,10,12,22].

## Conclusion

Currently, nurses have demonstrated a strong commitment to change and improvement in the health care services and systems in Iran. Nursing in Iran has been progressive as evidenced by comparing the history of nursing to where they are now. Therefore, empowerment is essential for enhancing nurses' role, strengthening the professional image, and continuously improving the healthcare system nationally and globally. Restructuring nursing services will eliminate barriers to poor quality nursing care, inadequate educational preparation, role ambiguity and low self-esteem among nurses. The nurses' experiences and their perceptions regarding empowerment were studied and a model was developed for empowerment of the nursing profession in Iran. This model was sent to the Ministry of Health and approval is pending. Once approved, the model will be implemented and applied in healthcare practice settings. This model will not only benefit Iranian nurse leaders, nurses, and The Ministry of Health, but nurses and leaders globally.

## List of abbreviations used

Baccalaureate Science in Nursing (BSN), American Presbyterian Missionary Society (APMS), World Health Organization (WHO), United States of America (USA), Iranian Nursing Association (INA), Associate Degree of nursing program (ADN), Masters of Science in nursing (MSN), Iranian Nursing Organization (INO), senior nursing directors (SND), Scientific Committee for Defining Nursing and its Mission" (SCDNM), Committee for Revision in Nursing Education" (CRNE), Human Resources and Restructuring Committee" (HRRC),

## Competing interests

The author(s) declare that they have no competing interests.

## Authors' contributions

MAH: Initiation and design of the research, collection and analysis of the data and writing the paper. MS: Co-analysis of the data and revision of draft paper.

## Pre-publication history

The pre-publication history for this paper can be accessed here:


